# Comment on: “Microplastic presence in dog and human testis and its potential association with sperm count and weights of testis and epididymis”

**DOI:** 10.1093/toxsci/kfae136

**Published:** 2024-10-30

**Authors:** Rao M Uppu, Willie Peijnenburg, Sean M Hays

**Affiliations:** Environmental Toxicology and Chemistry, College of Agriculture, Southern University and A&M College, Baton Rouge, LA 70807, United States; Center for Environmental Sciences, Leiden University, Leiden 2311 G, The Netherlands; SciPinion LLC, Bozeman, MT 59715, United States

The manuscript by Hu and coauthors provides what they claim is the presence of microplastic in dog and human testis. We respectfully raise issues with the conclusions drawn because it is our opinion that the analytical approach used is not robust enough to support these claims. We respectfully request the authors consider the following key concerns with this study.

First, there are no details on background contamination levels for sample collection, handling, and laboratory processing such as conducting sample blank(s), negative controls (e.g. procedural blanks), positive controls (e.g. spike-recovery), background controls (e.g. fume hood and laboratory spaces) tests, and their method detection and quantification limits (LOD/LOQ) to assess data accuracy (Hermsen et al., 2018; Brander et al. 2020). Without rigorous quality assurance and quality control, the risk of errors, contamination, or inaccuracies is increased and of unknown impact (Brander et al., 2020). Regarding sample collection and handling, insufficient details are provided on collection methods, storage conditions, and homogenization protocols. Potential contamination during surgical collection is not addressed, and long-term storage effects (up to 7 years for human samples) on sample integrity are not discussed. Without reporting any results for controls, one cannot assess whether the concentrations of microplastics reported for samples are real or not. They are a complete uncertainty and no way of knowing if false positives exist (Leslie et al. 2022).

Second, the digestion, homogenization, and ultracentrifugation steps were not validated, raising concerns about the potential loss or contamination of smaller particles (Kim et al. 2022 is a good example of careful validation procedures for the detection of quantification of microplastics). Additionally, no microscopic visualization or characterization of particle size and shape was performed, which is crucial for understanding potential biological interactions and toxicity (Garrido Gamarro and Costanzo 2022).

Third, the pyrolysis-gas chromatography/mass spectrometry (Py-GC/MS) method used lacks proper validation for analyzing microplastics in biological tissues (Leslie et al. 2022). The study also lacks information on precision, LOD/LOQ, and quantification methods and there is an absence of internal/surrogate standards and matrix spikes (Kirstein et al. 2021; Chand et al. 2022). Also, insufficient details are provided on Py-GC/MS parameters and setup. Furthermore, known challenges in detecting certain polymers like PVC and PET are not addressed, acknowledged, or mitigated (La Nasa et al. 2020; Kamp et al. 2023; Lauschke et al. 2023).

Despite these concerns, we want to emphasize that research on microplastics in human and animal tissues is of critical importance. While we acknowledge the importance of this research topic and the authors' efforts, it is our opinion that the methodological and analytical issues noted above potentially limits how Hu et al.’s (2024) findings should inform our understanding of the presence of microplastics in biological tissues. Early research in emerging fields often faces challenges, but it is crucial that published studies meet rigorous scientific standards to advance our understanding of this emerging health concern.

## Funding

Funding support for this article was provided by the American Chemistry Council. SciPinion received funding to conduct an independent peer review using a panel of independent experts. This letter to the editor captures the findings of the panel peer review.
